# Preparation and characterization of Sn/La co-doped TiO_2_ nanomaterials and their phase transformation and photocatalytic activity

**DOI:** 10.1038/s41598-018-30050-3

**Published:** 2018-08-17

**Authors:** Xiaodong Zhu, Lingxiu Pei, Ranran Zhu, Yu Jiao, Renyong Tang, Wei Feng

**Affiliations:** 1 College of Mechanical Engineering, Chengdu University, Chengdu, 610106 China; 2 College of Science, Xichang University, Xichang, 615013 China; 3 College of Materials and Chemistry & Chemical Engineering, Chengdu University of Technology, Chengdu, 610059 China; 4 Sichuan Engineering Research Center for Powder Metallurgy, Chengdu University, Chengdu, 610106 China; 5 College of Pharmacy and Biological Engineering, Chengdu University, Chengdu, 610106 China

## Abstract

The pure, tin (Sn)-doped, lanthanum (La)-doped and Sn/La co-doped titanium dioxide (TiO_2_) nanomaterials were synthesized using sol-gel method followed by calcination at the temperature of 360 °C, 450 °C and 600 °C, respectively. The structures of the nanomaterials were characterized by X-ray diffraction (XRD), Thermogravimetric (TG), Differential Thermal Analysis (DTA), Scanning Electron Microscopy (SEM), Energy Dispersive Spectrum (EDS), Transmission Electron Microscopy (TEM), X-ray Photoelectron Spectrum (XPS), Diffuse Reflectance Spectrum (DRS), Photoluminescence Spectrum (PL), Brunauer-Emmett-Teller Measurements (BET), respectively. The photocatalytic property of the photocatalysts under UV light was evaluated through the degradation of Rhodamine B (RhB). The results show that the anatase-rutile phase transition is promoted by Sn-doping while La-doping retards the phase transition. However, La doping plays a major role in the process of phase transformation. The photocatalytic activity of pure TiO_2_ is affected by annealing temperature remarkably and the optimal annealing temperature is 450 °C. The photocatalytic activity of TiO_2_ is enhanced significantly by Sn and La doping at three different temperatures. Sn/La-TiO_2_ exhibits the highest degradation rates and the fastest reaction rates probably owing to the synergistic effect of Sn^4+^ and La^3+^ ions in inhibiting the recombination of photogenerated electron-hole pairs. The formation of extra surface hydroxyl groups and additional surface area are also beneficial for the photocatalytic activity.

## Introduction

Environmental pollution is an increasingly serious problem, which restricts the development of economy severely. Photocatalytic technology is an effective way to solve the problem and several semiconductor photocatalysts have been used for decomposing organic pollutants such as zinc oxide^1^, cadmium sulfide^2^, carbon nanoparticle^3^, tungsten trioxide^4^ and so on. Among numerous candidate compounds, TiO_2_ has been widely used owing to its high photocatalytic activity, non-toxicity, low cost and stable chemical property^5–8^. However, pure TiO_2_ has two main faults such as low utilization of solar energy because of the wide band gap and low quantum yields since photogenerated electron-hole pairs recombine fast^9,10^. In order to improve the photocatalytic activity of pure TiO_2_, researchers adopt some strategies such as depositing with noble metals^11–13^, combining with other semiconductors^14–17^ and doping metal^18–20^ or nonmetal elements^21,22^.

Doping with La element boosts the photocatalytic activity of TiO_2_ efficiently because La ions are able to capture photogenerated electrons, prolong the lifetime of photogenerated electron-hole pairs and increase the thermostability of anatase phase which has higher photocatalytic activity than that of rutile phase^23,24^. Xin *et al*.^25^ reported that the discoloration rate of RhB was enhanced by La doping of the TiO_2_ photoelectrode. TiO_2_ doping with Sn is also an effective method to enhance photocatalytic activity since the coupling of TiO_2_ with SnO_2_ decreases the recombination of photogenerated electron-hole pairs and thus improves charge separation^20,26,27^. Bhange *et al*.^28^ have synthesized a series of Sn-doped TiO_2_ particles with different concentrations and photocatalytic tests show all the Sn-doped TiO_2_ have higher photocatalytic activity than that of pure TiO_2_. It is reasonable to suppose that doping with two dopants will lead to a synergistic effect and increase photocatalytic activity owing to their respective advantages. There are several reports about co-doped TiO_2_ which have proved that doping with two dopants shows higher photocatalytic activity than the single one^23,29–34^.

In the study of co-doped TiO_2_, most researchers focus on the concentration of dopants, however, research on the influence of heat treatment temperature is relatively few. It is generally known that heat treatment has a significant impact on the photocatalytic activity of TiO_2_ because crystalline phase and grain size of TiO_2_ are affected greatly by annealing temperature. Meanwhile, ion doping will impact phase structure and grain size evidently. The reports have mentioned that anatase-rutile phase transition will be restrained by La doping^25^ while it is promoted by Sn doping^26,28^. However, the phase transformation is not quite clear when Sn and La are doped together. Therefore, the purposes of this work was to synthesize Sn/La co-doped TiO_2_ nanomaterials and investigate their anatase-rutile phase transition as well as photocatalytic activities under UV light at different temperatures. For comparison, Sn-doped, La-doped and pure TiO_2_ nanomaterials were also prepared and investigated. For simplicity, Sn-doped, La-doped, and Sn/La co-doped TiO_2_ are labeled as Sn-TiO_2_, La-TiO_2_ and Sn/La-TiO_2_, respectively.

## Experiment

### **Preparation of pure and doped TiO**_**2**_**nanomaterials**

All the TiO_2_ nanomaterials were synthesized via a sol-gel route. In a typical synthesis of pure TiO_2_, solution A and B were prepared firstly. Solution A consisted of 15 mL tetrabutyl titanate and 30 mL absolute ethanol. Solution B was made from 5 mL deionized water, 7.5 mL acetic acid and 15 mL absolute ethanol. Solution B was added dropwise to solution A with vigorous stirring. The resulting sol was undergone aging for several hours to form gel. Afterwards, the gel was dried at 80 °C for 12 h and the resulting powders were annealed for 2 h at the temperature of 360 °C, 450 °C and 600 °C, respectively. The Sn-TiO_2_ and La-TiO_2_ were synthesized by the same procedure with certain amounts of SnCl_4_.5H_2_O or La(NO_3_)_3_.6H_2_O being added into solution B to obtain required doping concentration. The Sn/Ti molar ratios of Sn-TiO_2_ were 1%, 3%, 6%, 9% and the La/Ti molar ratios of La-TiO_2_ were 0.2%, 0.5%, 1%, 2%. Sn/La-TiO_2_ was prepared by the same way and the Sn/Ti, La/Ti molar ratios were 3% and 0.5%, respectively.

### Characterization

The crystal structures of the nanomaterials were analyzed using an X-ray diffractometer (XRD, DX-2700, China). Thermogravimetric and differential thermal analysis (TG-DTA) were performed using a thermal analyzer (STA409PC, Germany). Surface morphologies and element compositions were characterized by a field-emission scanning electron microscopy (FEI-Inspect F50, USA) equipped with an energy dispersive X-ray spectrometer (EDS). TEM and HRTEM images were obtained using a Tecnai G2 F20 transmission electron microscope with an acceleration voltage of 200 kV. X-ray photoelectron spectra (XPS) were recorded on a spectrometer (XSAM800, Britain) to examine the chemical states. UV-Vis diffuse reflectance spectra (DRS) were collected on a spectrophotometer (UV-3600, Japan). Photoluminescence (PL) spectra were measured by a luminescence spectrometer (F-4600, Japan) with a 150 W Xenon lamp as an excitation source. Surface areas were tested by using the Brunauer-Emmett-Teller (BET) theory to the nitrogen adsorption-desorption data.

### Photocatalytic activity measurement

The photocatalytic activity of the prepared nanomaterials under UV light was evaluated from the degradation of Rhodamine B (RhB). 300 mL 10 mg/L RhB solution was taken into a beaker as an objective degradation pollutant and 0.3 g TiO_2_ was added as photocatalysts. The mixed liquid was stirring for 30 min in dark to establish an adsorption-desorption equilibrium before illumination. It was then photo-irradiated by using a 250 W high-pressure mercury lamp (main emission wavelength 365 nm, light intensity 35 mW/cm^2^) as UV light source. The distance between lamp source and beaker was fixed at 10 cm. The change in the concentration of RhB was tested every 30 min by measuring absorbance at the maximum absorptive wavelength (553 nm) using a UV-Vis spectrophotometer (UV6100A, China) and the whole test lasted 180 min. The degradation rate (D) of RhB was calculated by equation: D = (A_0_ − A_t_)/A_0_, where A_0_ and A_t_ are the initial absorbance (at 553 nm) and absorbance at time “t”.

## Results and Discussion

### XRD analysis

**Figure 1:**
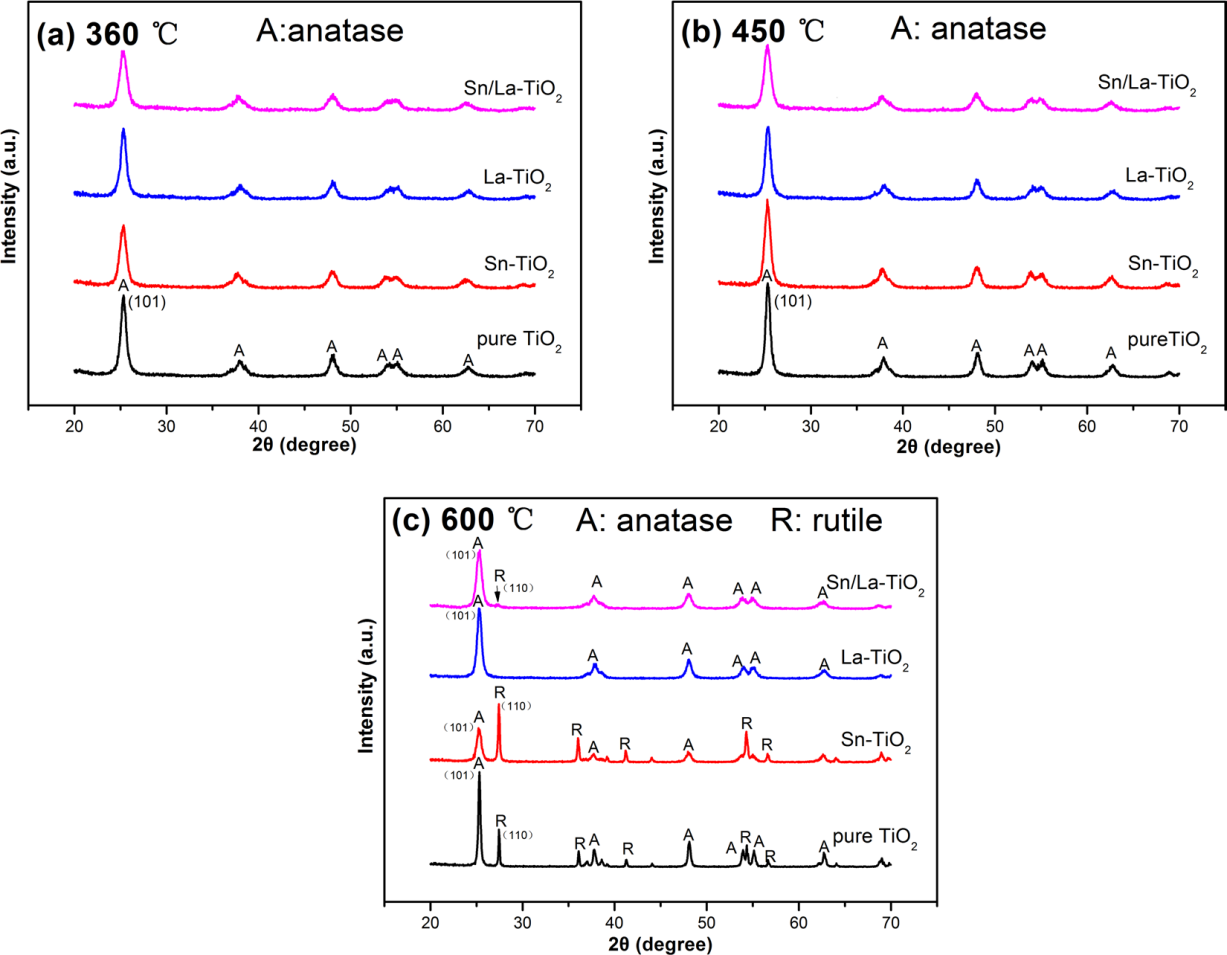
XRD patterns of pure TiO_2_, Sn-TiO_2_, La-TiO_2_ and Sn/La-TiO_2_ annealed at 360 °C (**a**), 450 °C (**b**) and 600 °C (**c**).

Figure 1 shows the XRD patterns of TiO_2_ nanomaterials at different temperature. All samples show peaks corresponding to anatase phase at the temperature of 360 °C and 450 °C. Compared to 360 °C, the intensity of peaks at 450 °C increases and the width of peaks becomes narrow, which imply that crystalline integrity improves with increase in temperature^19,35^. Pure TiO_2_ forms rutile phase when the heat treatment temperature is up to 600 °C. It is clear that the peak intensity of rutile phase of Sn-TiO_2_ is stronger than that of pure TiO_2_. However, there is no peak of rutile phase observed in La-TiO_2_, which indicates that anatase-rutile phase transition is promoted by Sn doping while it is suppressed by La doping. This is in agreement with the literature^25,26,28^. Ding *et al*.^36^ believe that the melting point of doped element oxides will affect anatase-rutile phase transition. The melting point of SnO_2_ is 1127 °C, which is lower than that of TiO_2_ (1640 °C), thus Sn as doping element promotes the transition. Meanwhile, the melting point of La_2_O_3_ is 2217 °C, which is higher than 1640 °C, as a result, La doping can retard the transition. As depicted in Fig. 1(c), a weak peak which corresponds to rutile (110) plane appears in the pattern of Sn/La-TiO_2_, indicates that the amount of rutile is less compared to pure TiO_2_. Obviously, the retarding effect by La doping plays a major role in the process of anatase-rutile phase transition although the dopant concentration of La (0.5%) is less than Sn (3%). The anatase/rutile weight ratios were calculated by the following equation^37^:

$${{\rm{X}}}_{{\rm{R}}}={(1+0.8({{\rm{I}}}_{{\rm{A}}}/{{\rm{I}}}_{{\rm{R}}}))}^{-1}$$

where X_R_ is the weight fraction of rutile, I_A_ and I_R_ are the relative strength of anatase (101) plane and rutile (110) plane, respectively. The average grain size (D) was calculated by Scherer’s formula^38^:

$${\rm{D}}=0.89\lambda /{\rm{\beta }}\,\cos \,{\rm{\theta }}$$

where λ is the wavelength of Cu Kα used, β is the full with at half maximum of the XRD peak and θ is the Bragg diffraction angle. The results are shown in Table 1. It clearly shows that the grain size of TiO_2_ decreases by doping and Sn/La-TiO_2_ presents the smallest grain size at the same temperature.

**Table 1:** Crystal phase and grain size of TiO_2_ nanomaterials.

Temperature	Samples	Crystal phase	Grain size/nm
360 °C	pure TiO_2_	Anatase	13.2
	Sn-TiO_2_	Anatase	11.5
	La-TiO_2_	Anatase	12.9
	Sn/La-TiO_2_	Anatase	11
450 °C	pure TiO_2_	Anatase	14.9
	Sn-TiO_2_	Anatase	13.2
	La- TiO_2_	Anatase	13.3
	Sn/La-TiO_2_	Anatase	11.3
600 °C	pure TiO_2_	Anatase (67.0%)/Rutile (33.0%)	29.3/51.2
	Sn-TiO_2_	Anatase (30.9%)/Rutile (69.1%)	15.9/39.8
	La-TiO_2_	Anatase	15.5
	Sn/La-TiO_2_	Anatase (95.8%)/Rutile (4.2%)	13.6/62.1

### TG-DTA analysis

**Figure 2:**
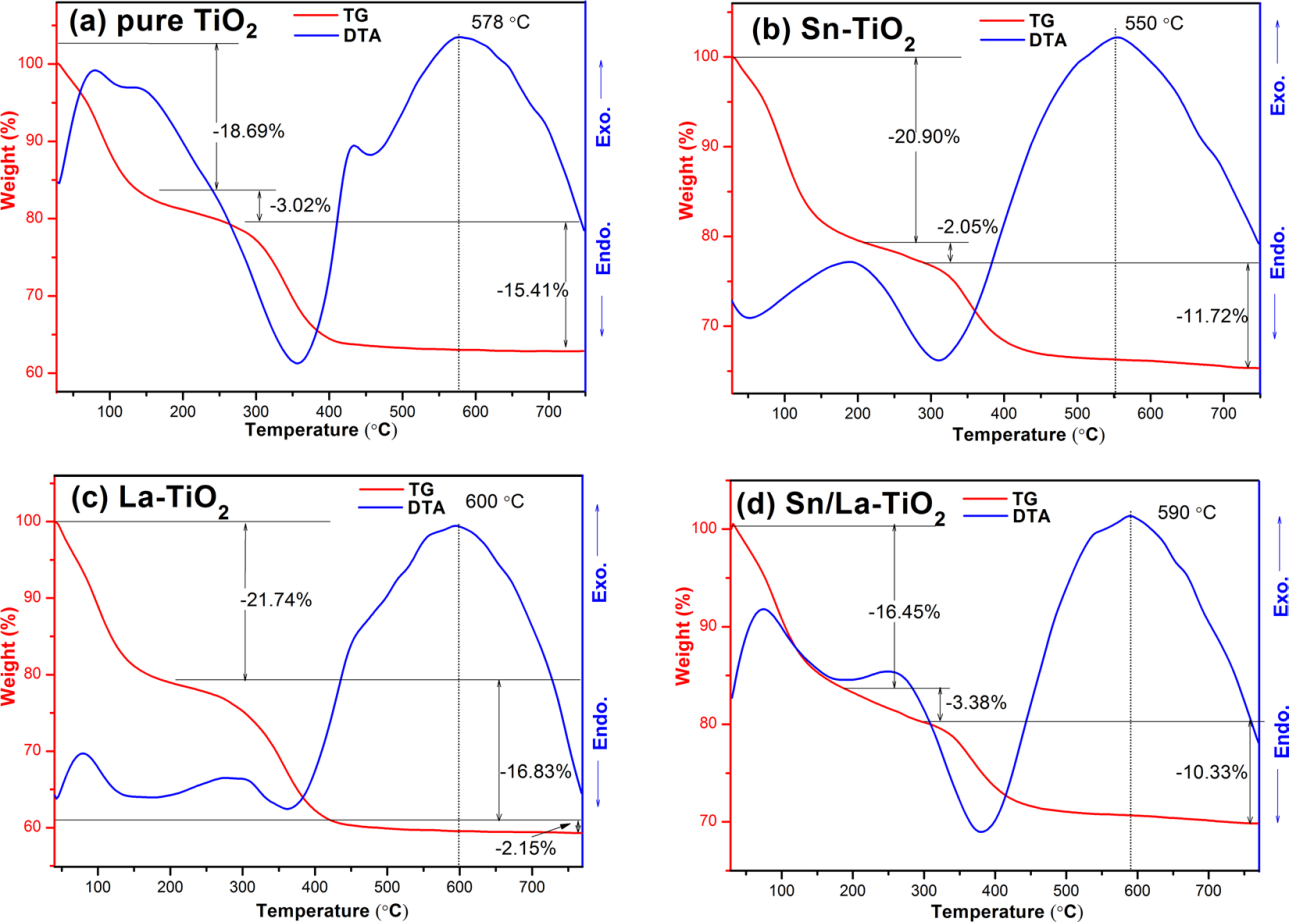
TG and DTA curves for pure TiO_2_ (**a**), Sn-TiO_2_ (**b**), La-TiO_2_ (**c**) and Sn/La-TiO_2_ (**d**).

Thermal analyses of pure TiO_2_ (a), Sn-TiO_2_ (b), La-TiO_2_ (c) and Sn/La-TiO_2_ (d) are depicted in Fig. 2. Left side of Y-axis represents the weight loss of samples. Right side of Y-axis represents exothermic and endothermic of samples during the heating process. The weight loss of all samples consists of three steps. In detail, the loss in the first step mainly derives from the evaporation of physically adsorbed water^26^. The combustion of organic compounds can be responsible for the second weight loss step. The third step of weight loss can be attributed to the dehydroxylation of the gel^31^. The exothermic peak at 578 °C in the DTA curve of pure TiO_2_ indicates that the phase transformation from anatase to rutile begin^5^. For Sn-TiO_2_ and La-TiO_2_, the exothermic peaks are approximately at 550 °C and 600 °C, respectively, which make clear that the phase transformation is promoted by Sn doping and is restrained by La doping. This is in accordance with XRD analysis and other literature^25,28^. It’s remarkable that the exothermic peak of Sn/La-TiO_2_ is approximately at 590 °C, suggesting that the phase transformation temperature of Sn/La-TiO_2_ is higher than that of pure TiO_2_. That means the La doping plays a leading role in the phase transformation process. This result also agrees well with XRD analysis.

### SEM and EDS analyses

**Figure 3:**
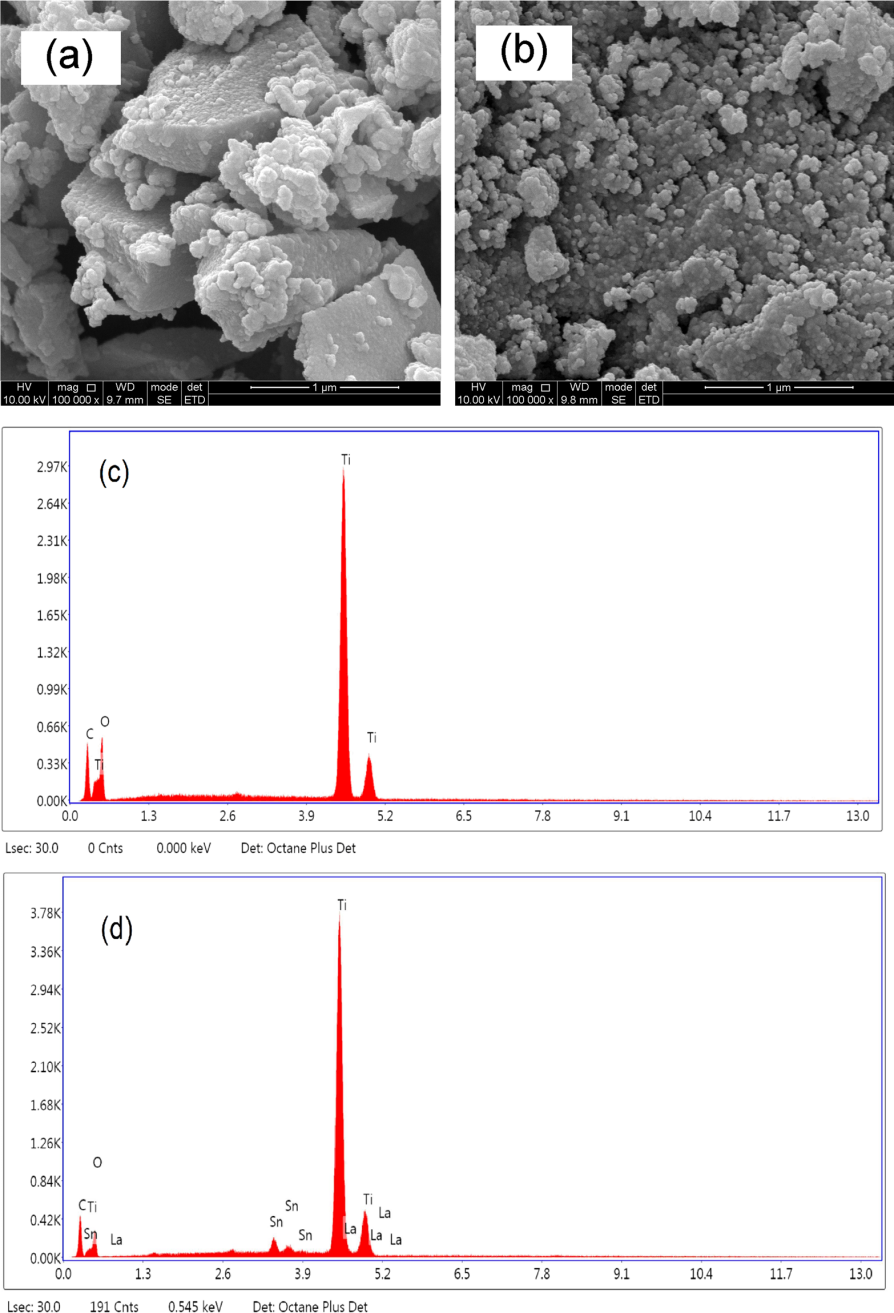
SEM images of pure TiO_2_ (**a**) and Sn/La-TiO_2_ (**b**) annealed at 450 °C and EDS spectra of pure TiO_2_ (**c**) and Sn/La-TiO_2_ (**d**) annealed at 450 °C.

Figure 3 presents the SEM images of pure TiO_2_ (a) and Sn/La-TiO_2_ (b) annealed at 450 °C. It is observed that pure TiO_2_ consists of agglomerated bulks which show different shapes and sizes. The diameter of aggregates in pure TiO_2_ ranges from 20–1000 nm. Sn/La-TiO_2_ presents relative uniform particles distribution and the particles show smaller size. The smaller particle size is favorable for the utilization of light source^39^.

EDS tests were carried out to examine the element composition of pure and Sn/La-TiO_2_ annealed at 450 °C and the results are shown in Fig. 3c,d. The peaks of C, Ti, O elements can be observed in the spectrum of pure TiO_2_. The peak of C derives from conductive plastic which was used in the EDS testing process. Except the peaks of C, Ti, O elements, the peaks of La and Sn elements appear in the spectrum of Sn/La-TiO_2_, which confirms that La and Sn elements exist in co-doped TiO_2_ nanomaterials.

### TEM analysis

**Figure 4:**
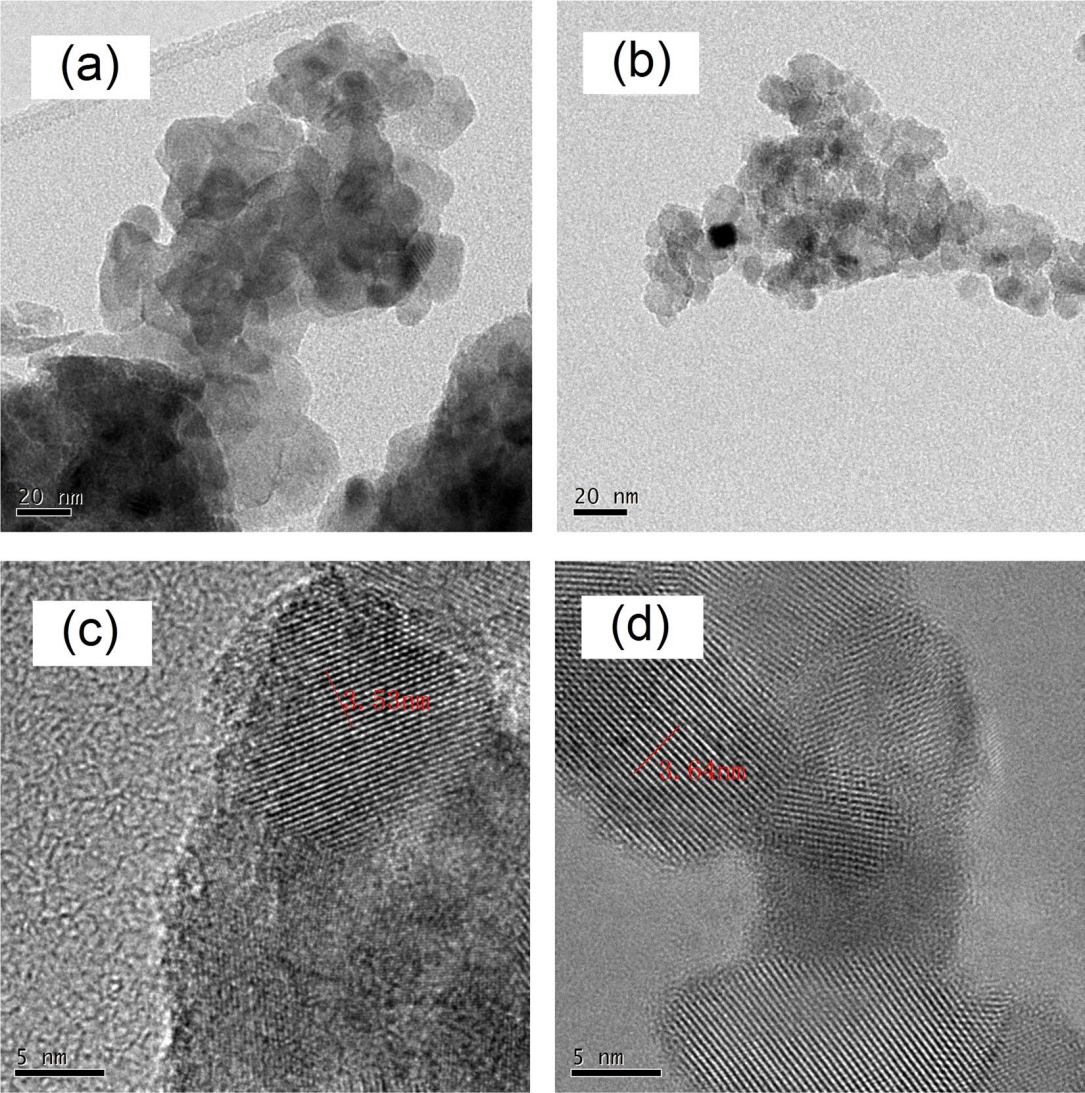
TEM images of pure TiO_2_ (**a**) and Sn/La-TiO_2_ (**b**) and HRTEM images of pure TiO_2_ (**c**) and Sn/La-TiO_2_ (**d**) annealed at 450 °C.

Figure 4 shows the TEM images of (a) pure TiO_2_ and (b) Sn/La-TiO_2_. It can be found that the average size of pure TiO_2_ is about 15 nm. Sn/La-TiO_2_ presents smaller size compared to pure TiO_2_, which is in accordance with the XRD results. In order to precisely investigate the structure of pure TiO_2_ and Sn/La-TiO_2_, further studies were carried out with a high-resolution transmission electron microscopy (HRTEM) and the images of pure TiO_2_ and Sn/La-TiO_2_ are shown in Fig. 4(c,d), respectively. The lattice fringes are clearly observed, which means that both pure TiO_2_ and Sn/La-TiO_2_ nanocrystalline form with good crystallinity^40^. The visible fringe spacings are 0.353 nm in Fig. 4(c) and 0.364 nm in Fig. 4(d), which can be assigned to the (101) plane of anatase TiO_2_. The (101) plane spacing of Sn/La-TiO_2_ increases due to the fact that the radius of Sn^4+^ (0.069 nm) is larger than that of Ti^4+^ (0.0605 nm), which indicates that Sn^4+^ ions have entered into TiO_2_ lattices^27^.

### XPS analysis

**Figure 5:**
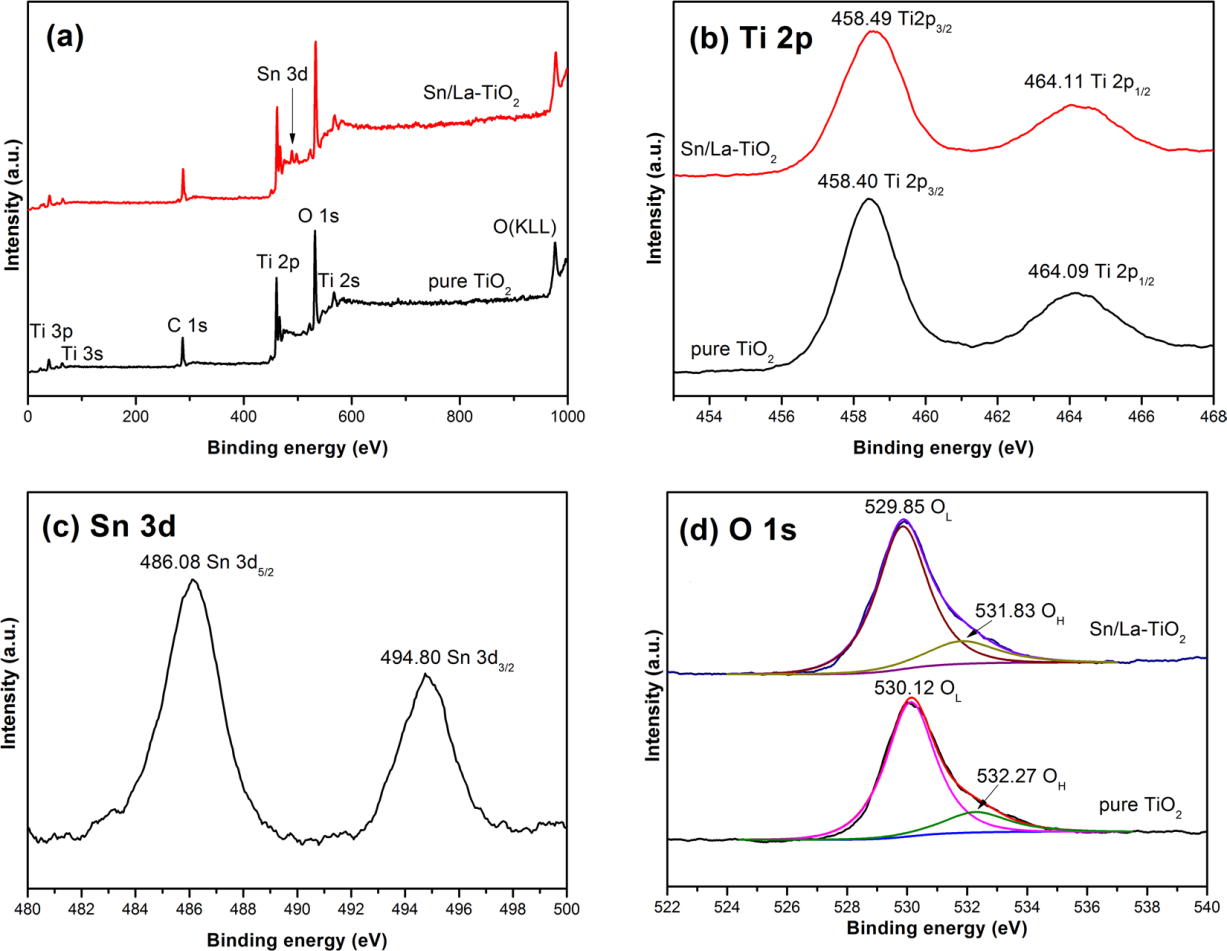
XPS spectra of pure TiO_2_ and Sn/La-TiO_2_ annealed at 450 °C. Total spectra (**a**), high resolution spectra of Ti 2p (**b**), high resolution spectrum of Sn 3d (**c**) and high resolution spectra of O 1 s (**d**).

In order to confirm the chemical states of elements in pure TiO_2_ and Sn/La-TiO_2_ annealed at 450 °C, XPS analysis was further carried out and the results are shown in Fig. 5. Figure 5(a) is the total spectra, which demonstrates that both pure TiO_2_ and Sn/La-TiO_2_ contain C, Ti, O elements. Besides, the peak of Sn 3d appears in the pattern of Sn/La-TiO_2_, indicating that Sn exists in TiO_2_ sample by doping. The absence of La element is probably because the low content of La is below the limit of XPS detection. The C 1 s peaks are mainly attributed to the oil pollution from equipment.

The high-resolution spectra of Ti 2p of pure TiO_2_ and Sn/La-TiO_2_ are shown in Fig. 5(b). It can be clearly observed that Ti 2p of pure TiO_2_ consists of two peaks at 458.40 eV and 464.09 eV, which correspond to Ti 2p_3/2_ and Ti 2p_1/2_, respectively. The slitting between Ti 2p_3/2_ and Ti 2p_1/2_ is 5.69 eV, which is convinced that Ti element exists in the form of Ti^4+^. Similar to pure TiO_2_, Ti 2p of Sn/La-TiO_2_ also consists of Ti 2p_3/2_ and Ti 2p_1/2_ peaks, which are located at 458.49 eV and 464.11 eV, respectively. The slitting of these two peaks is 5.62 eV, that means Ti element is also +4 state in Sn/La-TiO_2_^41,42^.

Figure 5(c) shows the high-resolution of Sn 3d spectrum. The peaks at 486.08 eV and 494.80 eV ascribe to Sn 3d_5/2_ and Sn 3d_3/2_, respectively, indicating that Sn element exists as Sn^4+^ ^27,28^.

The high-resolution spectra of O 1 s of pure TiO_2_ and Sn/La-TiO_2_ are shown in Fig. 5(d). The peaks of pure TiO_2_ at 529.85 eV and 531.83 eV can be attributed to lattice oxygen (O^2−^) and surface hydroxyl groups (OH^−^), respectively^11,35^. Meanwhile, the peaks of lattice oxygen and surface hydroxyl groups for Sn/La-TiO_2_ are at 530.12 eV and 532.27 eV. It is interesting to note that the proportion of surface hydroxyl groups in Sn/La-TiO_2_ is 18.1%, which is higher than that of pure TiO_2_ (16.6%). The extra surface hydroxyl groups are profitable to photocatalytic activity because the surface hydroxyl groups can be transformed into hydroxyl radicals, which decompose dye molecules effectively^11^.

### DRS analysis

**Figure 6:**
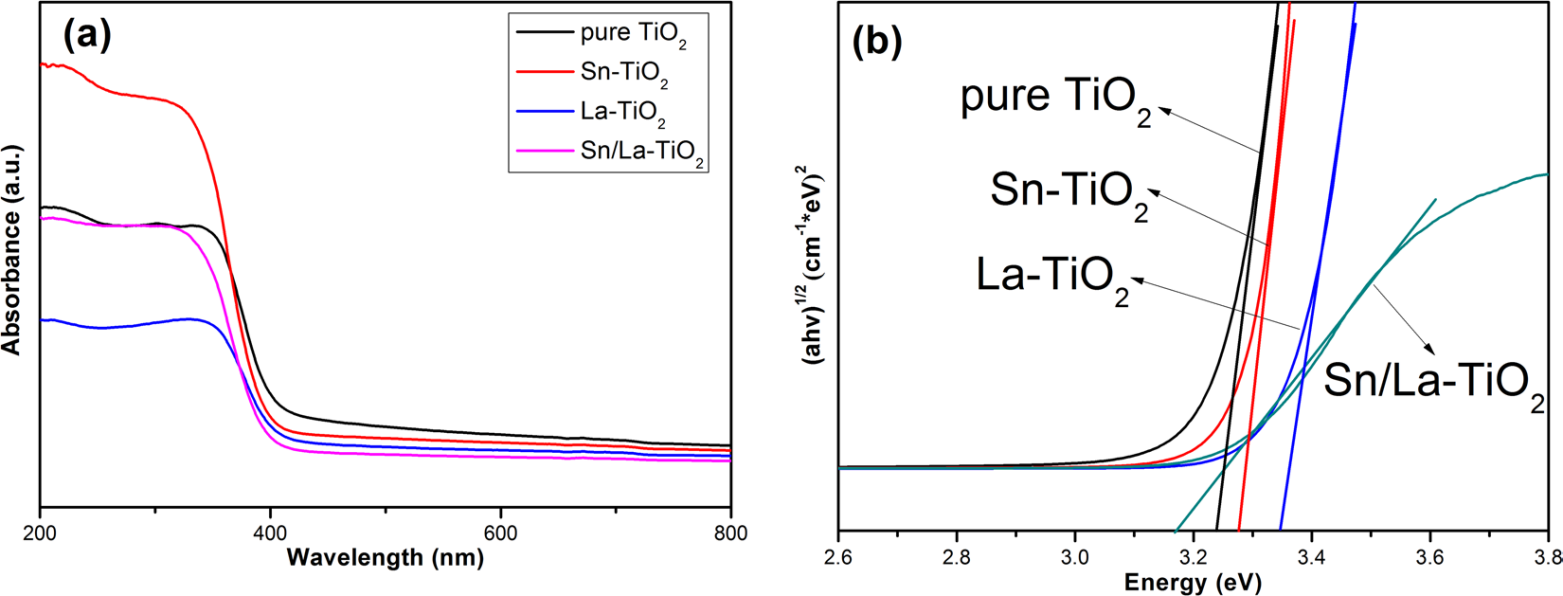
Diffuse reflection spectra of pure TiO_2_, Sn-TiO_2_, La-TiO_2_ and Sn/La-TiO_2_ annealed at 450 °C (**a**); plots of (αhv)^1/2^ versus the photon energy (hv) (**b**).

Figure 6 shows the DRS spectra of pure TiO_2_, Sn-TiO_2_, La-TiO_2_ and Sn/La-TiO_2_ calcined at 450 °C. The band gap energy (Eg) of samples can be calculated by the equation:

$${({\rm{\alpha }}{\rm{h}}{\rm{\nu }})}^{1/2}={\rm{A}}\,({\rm{h}}{\rm{\nu }}-{\rm{Eg}}),$$

where α is the absorption coefficient, hν is the photon energy and A is a constant^6^. The band gap energy of pure TiO_2_, Sn-TiO_2_, La-TiO_2_ and Sn/La-TiO_2_ are determined to be 3.22 eV, 3.27 eV, 3.35 eV and 3.17 eV, respectively. Sn or La as the doping elements always lead to red shift of TiO_2_ as reported before^27,43^, however, in this work, Sn-TiO_2_ and La-TiO_2_ show faint blue shift compared to pure TiO_2_. Bhange *et al*.^28^ believe that the blue shift is not owing to the quantum size effect since the grain size of TiO_2_ is larger than 10 nm, but is the result of the interaction between doping elements and TiO_2_ lattice and the formation of new energy levels. Sn/La-TiO_2_ shows slight red shift, which proves that the absorption of light improves by Sn and La co-doping.

### PL analysis

**Figure 7:**
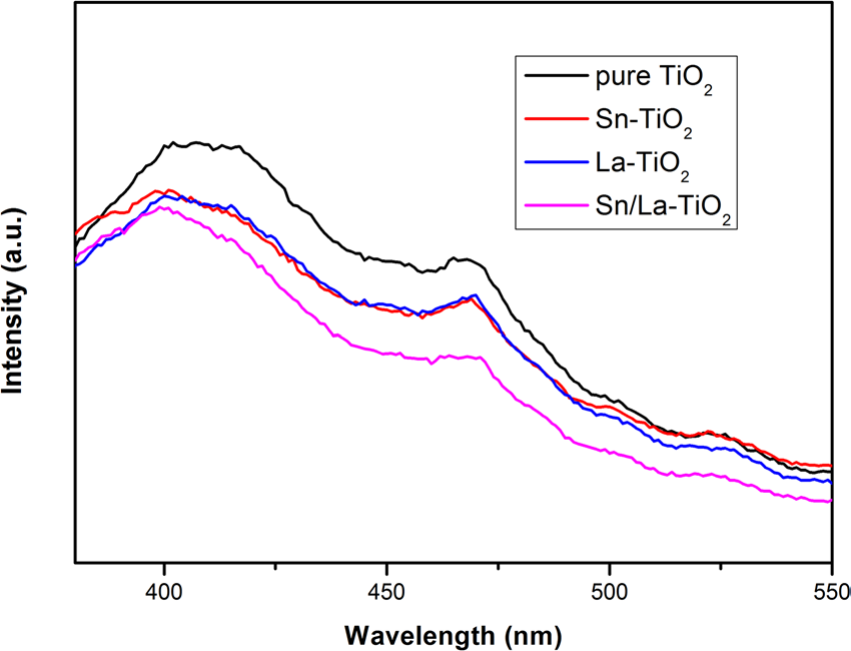
Photoluminescence spectra of pure TiO_2_, Sn-TiO_2_, La-TiO_2_ and Sn/La-TiO_2_ annealed at 450 °C.

It is well known that photoluminescence spectrum results from the recombination of photogenerated electrons and holes^29,31,35^. Therefore, in order to investigate the recombination and separation of photogenerated pairs, photoluminescence measurement was carried out and the results are shown in Fig. 7. The PL spectra of pure TiO_2_, Sn-TiO_2_, La-TiO_2_ and Sn/La-TiO_2_ calcined at 450 °C exhibit similar shape, however, the intensities of the samples are different. Pure TiO_2_ shows the maximal PL intensity compared to doped TiO_2_, suggesting that the recombination of photogenerated electrons and holes is restrained through Sn or La doping. It is noteworthy that the PL intensity of Sn/La-TiO_2_ is minimal, which indicates that Sn/La-TiO_2_ possesses the minimal recombination rate and the maximum separation rate of photogenerated pairs. The higher separation rate is beneficial to photocatalytic activity.

### BET analysis

**Figure 8:**
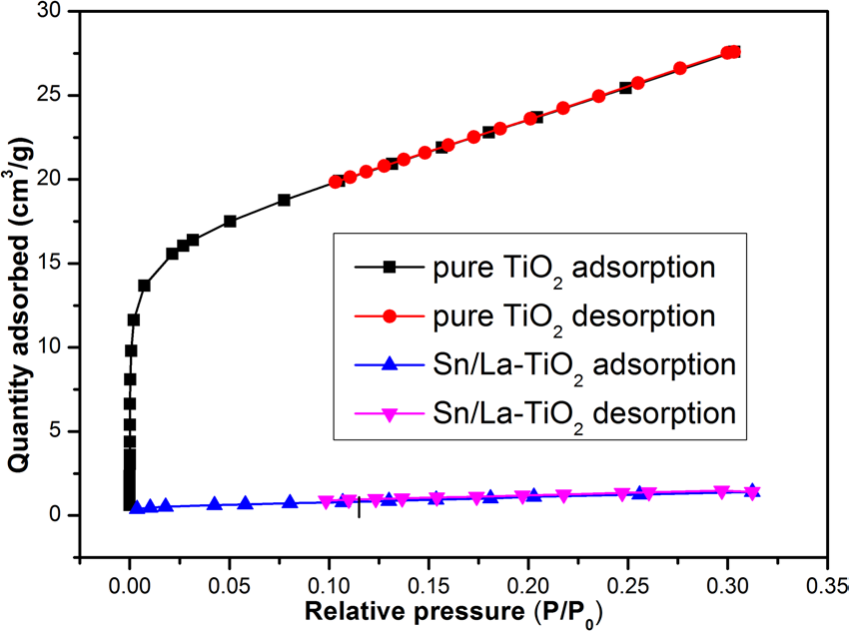
Nitrogen adsorption-desorption isotherm curves of pure TiO_2_ and Sn/La-TiO_2_ annealed at 450 °C.

From SEM images results, it is obvious that the aggregation is relieved and the particle size is decreased by Sn and La co-doping. In order to verify that the surface area of TiO_2_ is increased after co-doping, nitrogen adsorption-desorption isotherm measurement along with BET measurement is carried out and the N_2_ adsorption-desorption isotherms of pure TiO_2_ and Sn/La-TiO_2_ calcined at 450 °C are shown in Fig. 8. The BET specific surface areas of pure TiO_2_ and Sn/La-TiO_2_ are determined to be 4.4 m^2^.g^−1^ and 85.7 m^2^.g^−1^, respectively. The results manifest that Sn/La-TiO_2_ exhibits higher surface area than pure TiO_2_. The higher surface area is beneficial for the utilization of light source, which is able to improve photocatalytic activity^32^.

### Photocatalytic activity

**Figure 9:**
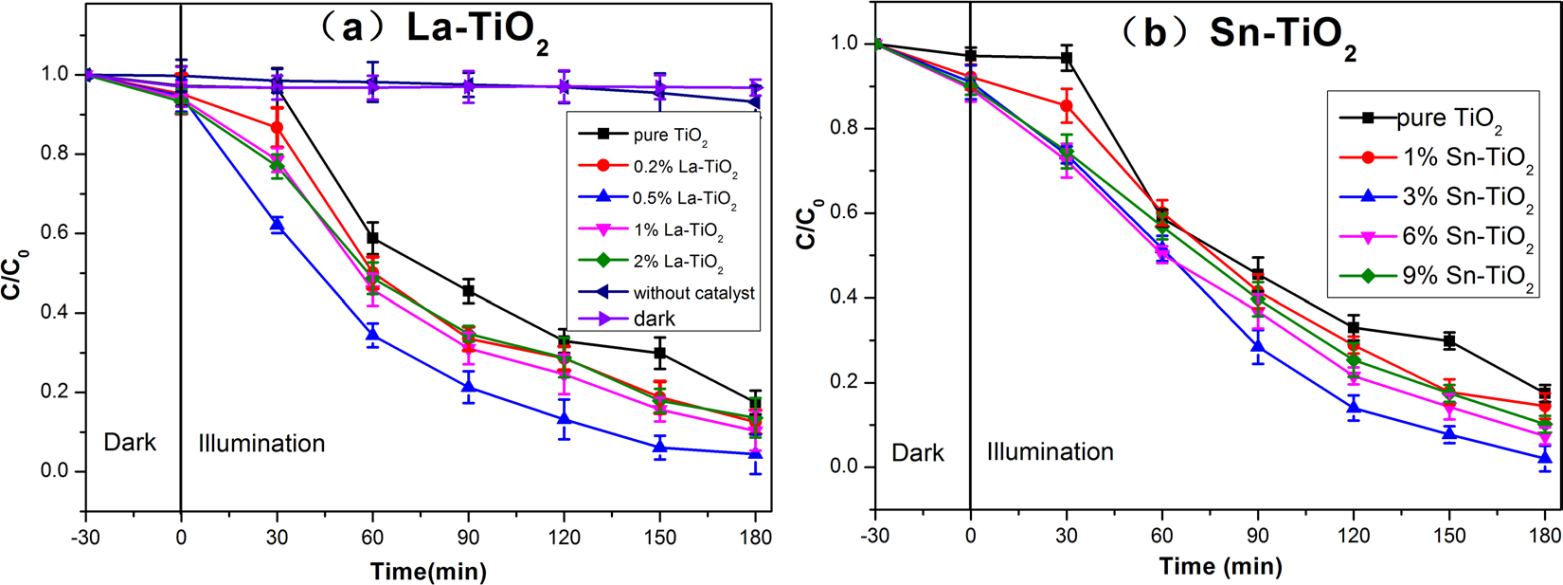
Photocatalytic degradation of RhB using (**a**) La-TiO_2_ and (**b**) Sn-TiO_2_.

The degradation of RhB was employed to evaluate the photocatalytic activity of the prepared TiO_2_ photocatalysts. Figure 9 shows the photocatalytic activity of Sn-TiO_2_ and La-TiO_2_ with different contents annealed at 450 °C.

The adsorption of RhB molecules was investigated via testing the degradation of RhB with catalyst adding under dark condition. The degradation rate of RhB is 3.2% on the condition of pure TiO_2_ adding in darkness, which is ascribed to the adsorption of RhB molecules on the surface of TiO_2_^44^. The self degradation of RhB without photocatalyst is 7%, which is negligible in the evaluation. Therefore, the degradation of RhB mainly is derived from the presence of photocatalysts under irradiation.

**Figure 10:**
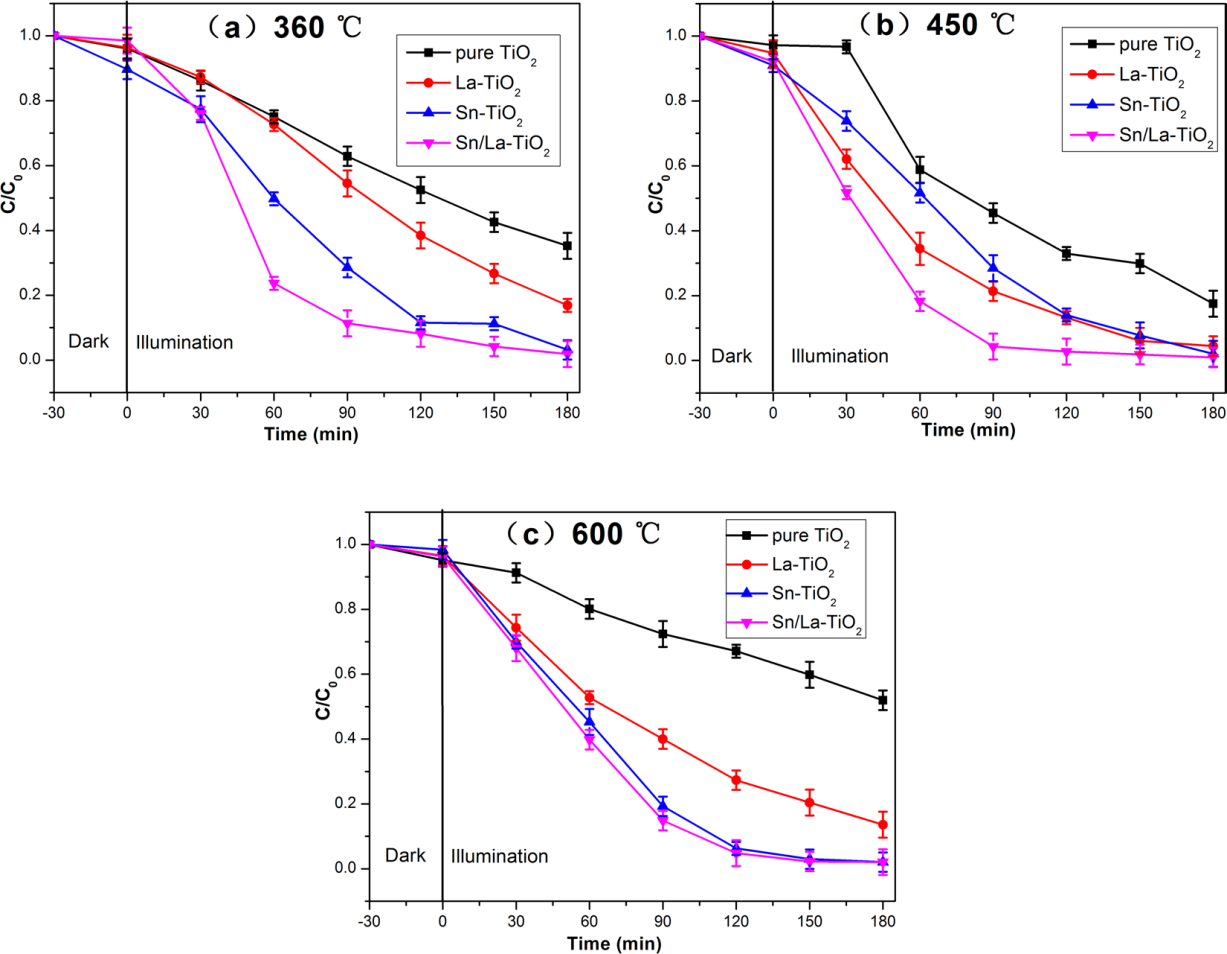
Photocatalytic degradation of RhB for pure TiO_2_, Sn-TiO_2_, La-TiO_2_ and Sn/La-TiO_2_ annealed at 360 °C (**a**), 450 °C (**b**) and 600 °C (**c**).

The degradation rate of pure TiO_2_ is 82.5% and all of the doped TiO_2_ show higher degradation rates. The optimal doping concentration for La-TiO_2_ is 0.5% and for Sn-TiO_2_ is 3%. Therefore, the molar ratios of Sn/La-TiO_2_ are determined to be La 0.5% and Sn 3%. The degradation rates of pure TiO_2_, Sn-TiO_2_, La-TiO_2_ and Sn/La-TiO_2_ annealed at 360 °C, 450 °C and 600 °C are shown in Fig. 10.

The degradation rates of pure TiO_2_ annealed at 360 °C, 450 °C and 600 °C are 64.7%, 82.5% and 48.0%, respectively. TiO_2_ annealed at 450 °C possesses the best photocatalytic activity than that of 360 °C and 600 °C. As discussed in XRD analysis, pure TiO_2_ annealed at 450 °C shows better crystalline integrity of anatase phase compared to 360 °C, which is positive for photocatalytic activity^45^. Meanwhile, pure TiO_2_ forms rutile phase at 600 °C and the high rutile content leads to poor photocatalytic activity^46,47^. Significantly, all of the doped TiO_2_ photocatalysts exhibit higher photocatalytic performance than pure TiO_2_ at three temperatures. The degradation rates of RhB for Sn/La-TiO_2_ are 98.1%, 99.1% and 97.9% at the annealing temperatures of 360 °C, 450 °C and 600 °C, which are 1.52 times, 1.20 times, 2.04 times higher than that of pure TiO_2_.

From XRD analysis, it can be noted that there is no peak for Sn, La and their oxides. Since the radius of La^3+^ (0.1061 nm) is much bigger than that of Ti^4+^ ion (0.0605 nm). Therefore, it is hard for La^3+^ ions to substitute Ti^4+^ ions in TiO_2_ lattice and it is reasonable to suppose that La^3+^ ions is located at the surface of TiO_2_^23,24,48^. La^3+^ ions act charge carrier trapping centers on the surface of TiO_2_, which suppresses the recombination of photogenerated electron-hole pairs and prolongs the lifetime, thus boosts the photocatalytic activity^23,49^. On the other hand, as the radius of Sn^4+^ ion is close to Ti^4+^ ion, Sn^4+^ ions are likely to substitute Ti^4+^ ions in the lattice of TiO_2_ and causes lattice distortion, which creates more surface defects. The surface defects are beneficial for the formation of oxygen vacancies and generating more superoxide radicals which degrade RhB molecules effectively^20,27,28^. Compared to pure and single element doped TiO_2_, Sn/La-TiO_2_ shows the highest photocatalytic activities at three temperatures because both Sn and La could enhance the photocatalytic activity as discussed before and Sn-La co-doping produces a synergistic effect. Moreover, from the results of XPS analysis, it is clear that the surface hydroxyl groups increase via Sn, La co-doping, which is propitious to promote photocatalytic activity. Besides, more surface area caused by Sn, La co-doping is also in favor of the enhancement of photocatalytic activity^23,27^.

**Figure 11:**
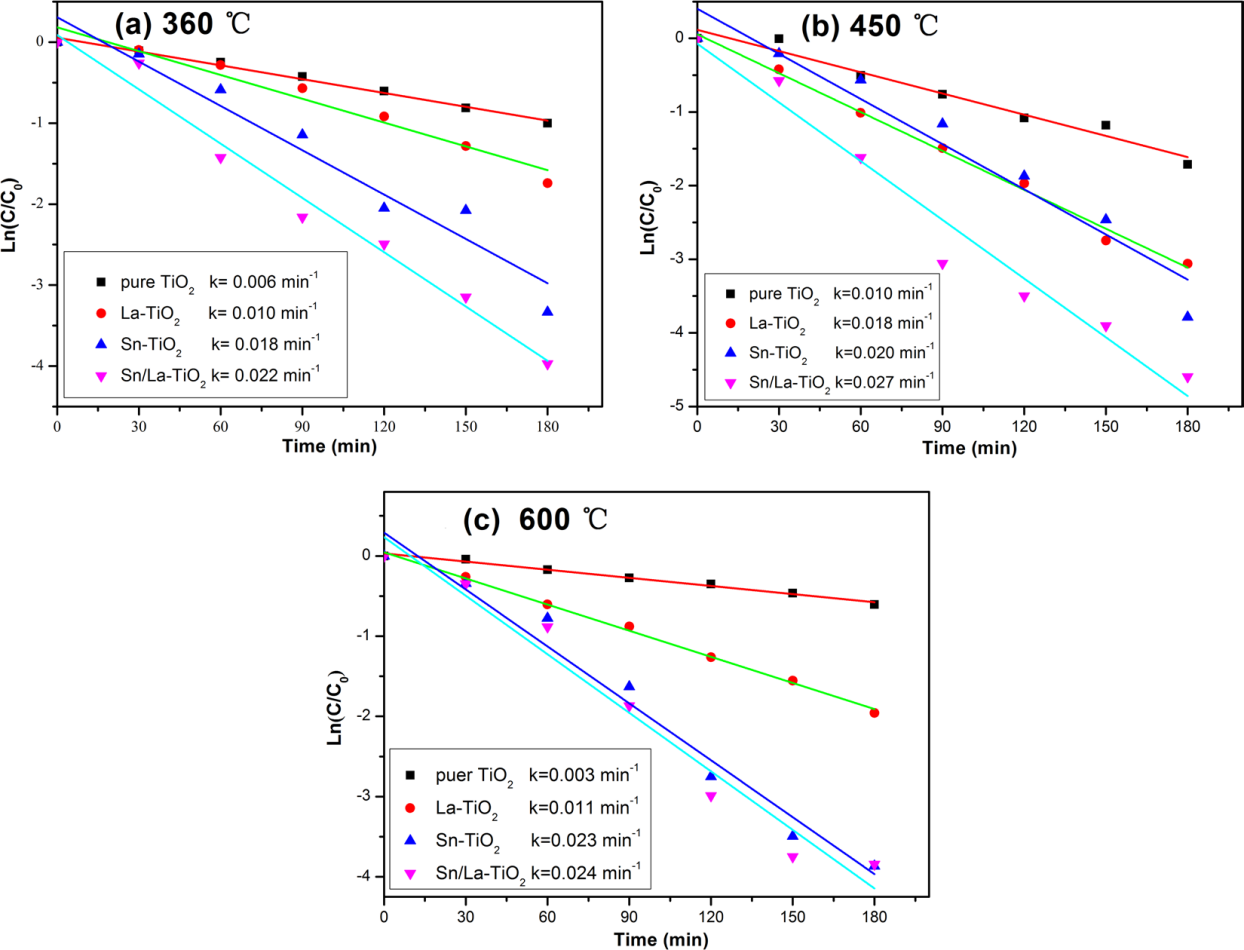
First-order reaction rate constants k against reaction time for different photocatalysts annealed at 360 °C (**a**), 450 °C (**b**) and 600 °C (**c**).

Figure 11 presents the kinetic fitting plots of ln (C/C_0_) against irradiation time t for the photocatalysts, which show linear relationships, indicating that the degradation of RhB by TiO_2_ photocatalysts follows pseudo first order kinetics^41^. The apparent reaction rate constant k is calculated by:

$$\mathrm{ln}\,({\rm{C}}/{{\rm{C}}}_{0})=-\,{\rm{k}}\,{\rm{t}}$$

where t is the reaction time, C and C_0_ are the concentration at time t and the initial concentration of RhB solution, respectively.

**Figure 12:**
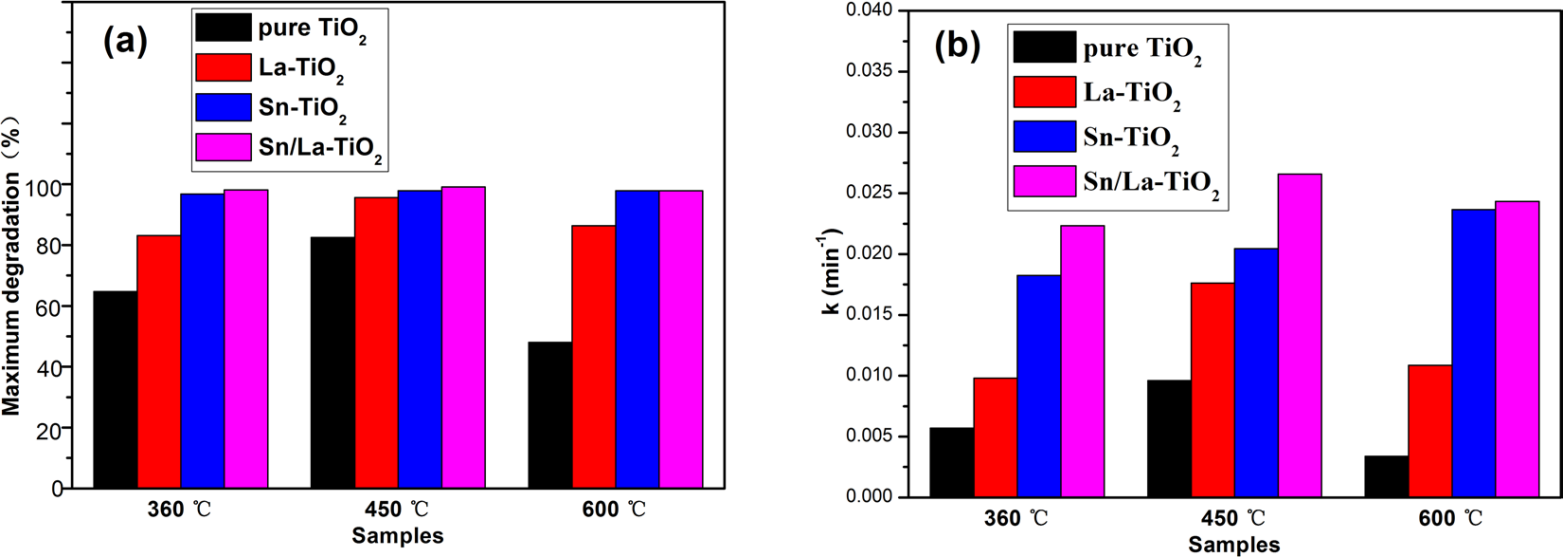
Comparison charts of degradation rates (**a**) and reaction rate constants k (**b**) for all of the photocatalysts.

The ultimate degradation rates after 180 min and the apparent reaction rate constant k of all the photocatalysts are shown in Fig. 12 for a better comparison. The Sn/La-TiO_2_ also exhibit the fastest reaction rate and the apparent reaction rate constant k are 0.022 min^−1^, 0.027 min^−1^ and 0.024 min^−1^ at 360 °C, 450 °C and 600 °C, which are 3.7 times, 2.7 times and 8 times more than that of pure TiO_2_. The photocatalytic performance of TiO_2_ can be enhanced remarkably by Sn and La co-doping and the most noticeable enhancement effect occurs at the heat treatment temperature of 600 °C. The degradation data for all of the catalysts are shown in Table 2.

**Table 2:** Degradation rates of RhB (a) and reaction rate constants k (b) of photocatalysts.

Temperature	Samples	Degradation rate	k (min^−1^)	R^2^
360 °C	pure TiO_2_	64.7%	0.006	0.989
	Sn-TiO_2_	96.8%	0.018	0.938
	La-TiO_2_	83.1%	0.010	0.954
	Sn/La-TiO_2_	98.1%	0.022	0.980
450 °C	pure TiO_2_	82.5%	0.010	0.962
	Sn-TiO_2_	97.9%	0.020	0.932
	La-TiO_2_	95.6%	0.018	0.993
	Sn/La-TiO_2_	99.1%	0.027	0.961
600 °C	pure TiO_2_	48.0%	0.003	0.986
	Sn-TiO_2_	97.9%	0.023	0.970
	La-TiO_2_	86.4%	0.011	0.997
	Sn/La-TiO_2_	97.9%	0.024	0.961

In order to study the effect of catalyst dosage, temperature, pH value and recyclability on the photocatalytic activity of catalyst, pure TiO_2_ annealed at 450 °C was chosen for the experiments.

**Figure 13:**
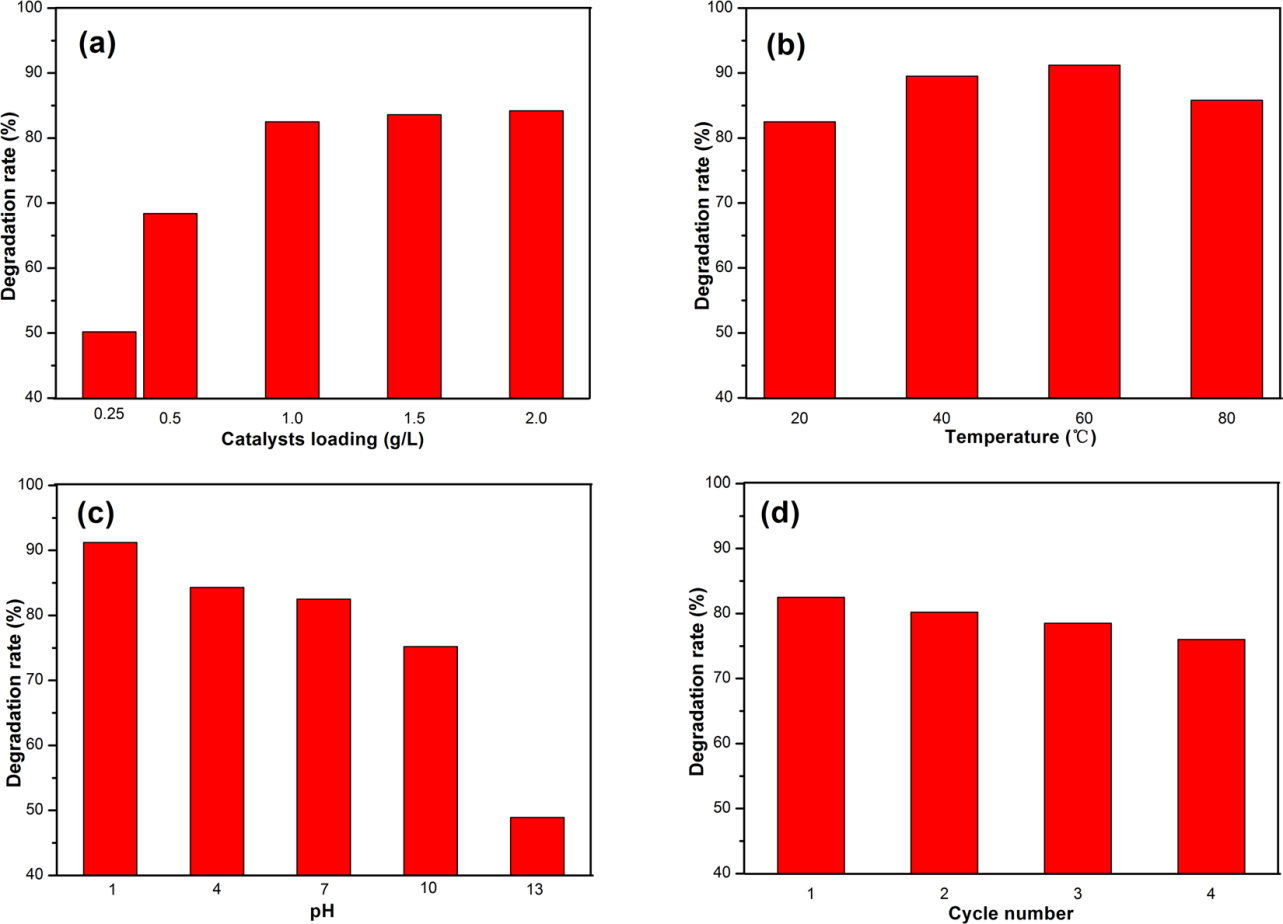
The effect of catalyst dosage, temperature, pH value and recyclability on the photocatalytic activity of catalyst.

In Fig. 13(a), the comparison of the degradation rate obtained with 0.25 g/L, 0. 5 g/L, 1 g/L, 1.5 g/L and 2 g/L catalyst is shown. The degradation rate increases with raising catalyst amount and the enhancement of degradation rate can be attributed to more surface area^50^. However, the degradation rate increases slightly when the concentration of catalyst surpasses 1 g/L. The aggregation of TiO_2_ particles occurs when the catalyst dosage is at high level, which leads to a decrease in surface area^51^.

The effect of temperature on the degradation rate of catalyst was studied by performed the photocatalytic experiments under temperatures ranging from 20 °C to 80 °C and the results are shown in Fig. 13(b). The degradation rare first increases when the temperature reach 60 °C and then decreases when the temperature is up to 80 °C. The oxidation rate of RhB molecules enhances with increase in temperature^50^. However, more rising the temperature is harmful to the photocatalytic activity because the recombination rate of photogenerated pairs and the desorption process of RhB molecules increase^51^.

In order to study the impact of the pH value on the degradation of RhB by catalyst, RhB solution was adjusted to different pH value (1, 4, 7, 10 and 13) by HCl (1 mol/L) and NaOH (1 mol/L). The results in Fig. 13(c) show that the degradation rate can be changed by varying the pH value. Acid environment shows higher degradation rate than neutral and alkaline environment. The PZC of pure TiO_2_ is about 6.25, therefore, the adsorption on catalyst is poor in alkaline solution. Acid solution is beneficial for reducing agglomeration and the formation of hydroxyl radicals, which lead to higher photocatalytic activity^50^.

The photocatalytic experiment was repeated four cycles to test the reusability of pure TiO_2_ catalyst and the results are shown in Fig. 13(d). The degradation rate after four cycles is 76.0%, which is slightly lower than that of the first cycle (82.5%). The decrease in the degradation rate can be attributed to the loss in adsorption between TiO_2_ catalyst and RhB molecules^44^.

**Figure 14:**
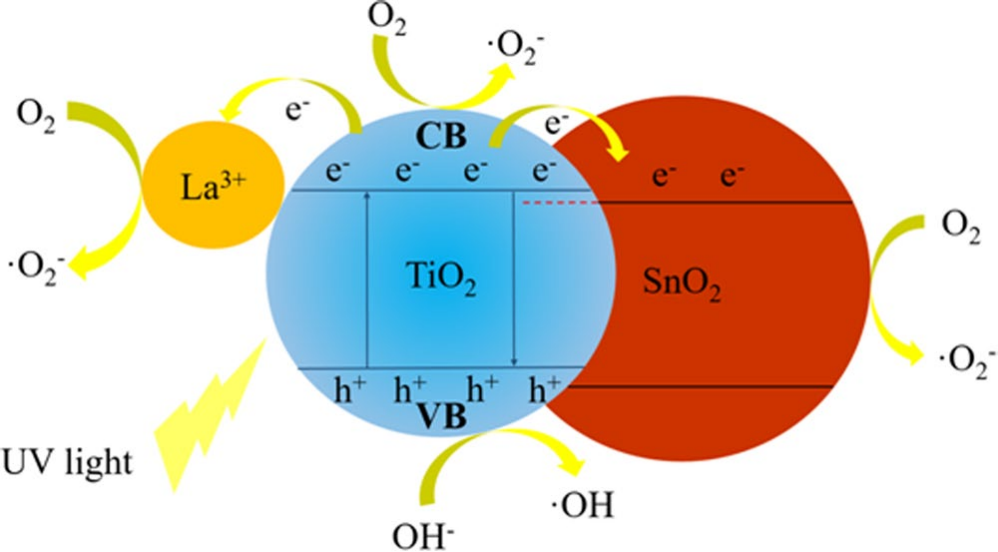
The schematic diagram of band structure and electron pairs separation in Sn/La-TiO_2_.

The schematic diagram of band structure and electron pairs separation in Sn/La-TiO_2_ is shown in Fig. 14. The electrons in valence band (VB) are excited to conduction band (CB) when TiO_2_ catalyst is irradiated by UV light, leaving holes in valence band^52^. On the one hand, La^3+^ ions which on the surface of TiO_2_ can trap the photogenerated electrons in conduction band, consequently, the recombination of electrons and pairs is retarded and the photogenerated pairs are separated effectively^47^. On the other hand, the photogenerated electrons in conduction band of TiO_2_ can be transferred to conduction band of SnO_2_, since the conduction band energy level of SnO_2_ is lower than that of TiO_2_^19,53,54^. Both Sn and La species are beneficial for the separation of photogenerated pairs, therefore, more electrons and holes can be involved into the photocatalytic degradation of RbB molecules. The holes combine with hydroxyl ions (OH^−^) to form hydroxyl radicals (.OH) and the electrons combine with adsorbed oxygen (O_2_) to form super-oxygen ions (.O_2_^−^)^3,54^. The resulting radicals degrade RbB molecules into smaller hydrocarbons and finally to CO_2_ and H_2_O^55^.

## Conclusions

In summary, pure TiO_2_, Sn-TiO_2_, La-TiO_2_ and Sn/La-TiO_2_ nanomaterials experienced different temperature heat treatment were successfully synthesized via sol-gel route. The photocatalytic activity of the prepared TiO_2_ photocatalysts under UV light were tested by the degradation of RhB. Calcination temperature has an obvious influence on the photocatalytic property of pure TiO_2_ and it shows the best degradation rate at 450 °C. Doping with Sn and La enhances the photocatalytic activity of TiO_2_ significantly at three temperatures and Sn/La-TiO_2_ exhibit the highest degradation rates. The effect of co-doping on the improvement of photocatalytic performance can be attributed to the synergistic effect in suppressing photogenerated pairs and the formation of extra surface hydroxyl groups and more surface area involved during the reaction process.
